# ITF6475, a New Histone Deacetylase 6 Inhibitor, Prevents Painful Neuropathy Induced by Paclitaxel

**DOI:** 10.3390/toxics13090767

**Published:** 2025-09-10

**Authors:** Guido Cavaletti, Annalisa Canta, Alessia Chiorazzi, Eleonora Pozzi, Valentina Carozzi, Cristina Meregalli, Paola Alberti, Paola Marmiroli, Arianna Scuteri, Luca Crippa, Silvia Fermi, Ibtihal Segmani, Barbara Vergani, Christian Steinkühler, Simonetta Andrea Licandro

**Affiliations:** 1 Experimental Neurology Unit, School of Medicine and Surgery, University of Milano-Bicocca, 20900 Monza, Italy; annalisa.canta@unimib.it (A.C.); alessia.chiorazzi@unimib.it (A.C.); eleonora.pozzi@unimib.it (E.P.); valentina.carozzi1@unimib.it (V.C.); cristina.meregalli@unimib.it (C.M.); paola.alberti@unimib.it (P.A.); paola.marmiroli@unimib.it (P.M.); arianna.scuteri@unimib.it (A.S.); luca.crippa@unimib.it (L.C.); silvia.fermi@unimib.it (S.F.); i.segmani@campus.unimib.it (I.S.); 2 Fondazione IRCCS San Gerardo dei Tintori di Monza, 20900 Monza, Italy; 3 Laboratory of Scientific and Clinical Research in Cancer Pathology, Faculty of Medicine and Pharmacy, University Hassan II of Casablanca, Casablanca 21100, Morocco; 4 Preclinical R&D Department, Italfarmaco S.p.A., 20092 Cinisello Balsamo, Italy; b.vergani@italfarmacogroup.com (B.V.); s.licandro@italfarmacogroup.com (S.A.L.)

**Keywords:** paclitaxel, neuropathy, histone deacetylase 6, neuroprotection, small fibers, ITF6475

## Abstract

Chemotherapy-induced peripheral neuropathy remains a significant side effect of cancer treatment, often requiring dose reductions or even discontinuation of therapy. Paclitaxel (PTX), a widely used chemotherapeutic agent for solid tumors, is particularly neurotoxic, and no effective treatment exists for paclitaxel-induced peripheral neuropathy (PIPN). Histone deacetylases (HDACs) are enzymes that remove acetyl groups from histone and non-histone proteins, including transcription factors and cytoskeletal components. This study evaluates the HDAC6 inhibitor ITF6475 for its potential to prevent PIPN and compares its effects with ricolinostat, a well-established HDAC6 inhibitor previously studied in cisplatin-induced neuropathy models. Female C57BL/6 mice received PTX vehicle (VEH) or PTX (70 mg/kg intravenously, once per week for four weeks), and the remaining four groups received PTX with co-treatment of either ricolinostat (50 mg/kg orally, daily) or ITF6475 (1, 6, or 12.5 mg/kg orally, daily). Neurophysiological assessments at the end of treatment showed a significant reduction in caudal sensory nerve action potential amplitude across all PTX-treated groups compared to the VEH group. At the same time, PTX treatment led to the development of mechanical allodynia. However, co-treatment with the HDAC6 inhibitor prevented significant differences compared to the VEH group. PTX-induced reduction in intraepidermal nerve fiber density was significantly prevented in the PTX + ITF6475 (1 mg/kg) group, and PTX-induced increase in neurofilament light levels was reduced in all ITF6475 co-treated groups. These findings support the potential of ITF6475 in preventing small fiber damage in a severe, chronic PIPN model.

## 1. Introduction

Improvement in the treatment of solid and hematological tumors significantly increased the long-term survival of cancer patients, but sometimes, these patients experience long-term treatment-related side effects that can have severe consequences on their quality of life [1,2,3,4,5,6]. Since several typical severe side effects of chemotherapy, such as anemia, neutropenia, nausea, and vomiting, can now be more effectively managed with specific cell growth factors or anti-emetics modulating specific molecules [6], new dose-limiting off-target toxic events have emerged. Chemotherapy-induced peripheral neuropathy (CIPN) emerged as a major side effect of cancer treatment, often necessitating dose reductions or even discontinuation of therapy [7]. This is particularly concerning not only because of its impact on patients’ health status, but also because it can compromise the effectiveness of anticancer treatments. In fact, in the absence of effective treatments to prevent or, at least, limit the severity of CIPN, neurotoxic chemotherapy drug dose reduction (or even withdrawal in the most severe cases) is the only available strategy to prevent irreversible peripheral nervous system damage. This uncomfortable and highly clinically relevant situation prompted several treatment and prevention attempts, mostly based on pharmacological approaches, but also on physical treatments (e.g., physiotherapy, hand/foot compression, cryotherapy, and acupuncture) [8,9,10,11]. Unfortunately, none of these attempts showed clear evidence of efficacy.

CIPN affects, with different clinical patterns and severity, patients with various common tumors, such as breast, lung, gastrointestinal, and prostate cancers, as well as rarer malignancies like testicular and ovarian cancers, and multiple myeloma. The incidence of CIPN can exceed 80% in patients, depending on the chemotherapy regimen used, and its symptoms are often irreversible, significantly diminishing patients’ quality of life [12].

The most commonly neurotoxic anticancer drugs have different antineoplastic mechanisms, including binding to nuclear DNA, inhibition of proteasomal activity, and disruption of the microtubular array. These mechanisms are not necessarily the same causing CIPN, but, particularly regarding the anti-tubulin agents, this might be the case. Paclitaxel (PTX), the first-in-class drug of the taxane family, is a widely used chemotherapeutic agent for solid tumors (particularly breast, ovary, lung, prostate, and head-and-neck cancers), acting by hyper-stabilizing cancer cell microtubules, which are essential structures for highly dividing cells, such as cancer cells. Through this mechanism, PTX promotes the assembly of tubulin dimers into stable microtubule bundles and inhibits their breakdown, thus disrupting the cell cycle. In fact, this tubulin stabilization leads to cancer cells’ mitotic arrest in the G2/M phase, ultimately causing cell death and preventing their replication.

PTX is very effective, but it is also particularly neurotoxic, causing severe axonopathy involving all types of nerve fibers (commonly named PTX-induced peripheral neuropathy, or PIPN), for which there is currently no effective treatment. While the exact mechanisms underlying PIPN are not fully understood, research suggests that mitochondrial dysfunction, oxidative stress, and microtubule aggregation—leading to cytoskeletal damage and impaired axonal transport—play key roles [13].

Histone deacetylases (HDACs) are enzymes responsible for removing acetyl groups from histone and non-histone proteins, including transcription factors and cytoskeletal components. HDAC6, a specific member of this family, deacetylates α-tubulin in microtubules, thereby regulating mitochondrial transport [14]. In vitro studies have shown that inhibiting HDAC6 with selective inhibitors increases α-tubulin acetylation and enhances mitochondrial transport along hippocampal neuron axons [15].

CIPN, as well as the use of HDACH6 as a putative neuroprotectant agent, has been extensively investigated using rodent animal models. Experimental research on CIPN induced by drugs such as cisplatin and vincristine, as well as studies on PIPN and inherited neuropathy models, suggest that HDAC6 inhibitors have neuroprotective effects [16,17,18,19,20,21,22,23]. This neuroprotection is likely due to the preservation of the axonal cytoskeleton [24].

Most known HDAC6 inhibitors are hydroxamic acids, which rely on the hydroxamic group for enzyme inhibition. However, these compounds present pharmacological challenges: their rapid metabolism leads to a short circulation time, limiting sustained target inhibition. Additionally, hydroxamic acid hydrolysis can release hydroxylamine, a genotoxic and potentially carcinogenic compound, making these molecules unsuitable for chronic neuropathy treatment [25].

To address these limitations, a novel class of non-hydroxamic, non-genotoxic HDAC6 inhibitors has been developed. These compounds exhibit high selectivity for HDAC6, reducing off-target effects. One such compound, ITF6475, has shown strong HDAC6 inhibition without toxicity in animal studies following both intravenous and oral administration [26].

In this study, ITF6475 was evaluated for its potential to prevent PIPN and was compared in a thoroughly characterized mouse model [27] with ricolinostat (also indicated as ACY1215), a well-established HDAC6 inhibitor previously tested in experimental cisplatin-induced neuropathy, another severe form of CIPN [22]. In our study, we selected a mouse model where PIPN is induced with a high dose of the neurotoxic agent administered with a schedule based on repeated dosing with 1-week intervals. This model demonstrated to be optimal in a head-to-head comparison with a less intense treatment schedule, not only when behavioral assessments are used as study readouts, but when pathological and neurophysiological evidence of PIPN is also searched, so that the capacity of ITF6475 and ricolinostat to act as a neuroprotectant could be tested on the full spectrum of neurotoxicities induced by PTX.

## 2. Materials and Methods

### 2.1. Drugs and Animal Model

The care and husbandry of animals were conducted in agreement with the institutional guidelines in compliance with national (D.L.vo n. 26/2014) and international laws and policies (EU Directive 2010/63/UE; Guide for the Care and Use of Laboratory Animals, U.S. National Research Council, 8th Ed.). According to the Italian laws and regulations, the study plan was assessed by the Superior Institute of Health and authorized by the Italian Ministry of Health (authorization number 777/2022-PR).

A paclitaxel (LC Laboratories, Woburn, MA, USA) solution was prepared before each administration; the powder was dissolved in Tween80 10%, EtOH100 10%, and saline solution 80% and administered intravenously (i.v.). Ricolinostat and ITF6475 solutions were prepared before each administration, respectively, in DMSO 5%, PEG400/H2O 50/50, and methocel 0.5%, and administered orally (p.o).

**Figure 1:**
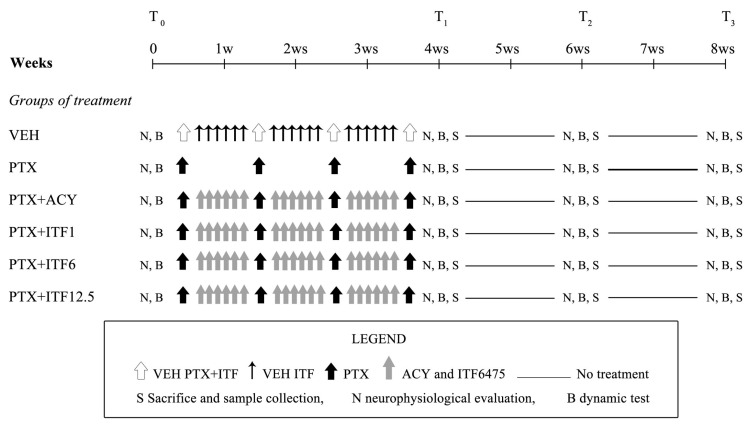
Flow chart of the study.

C57BL/6 female mice (Inotiv, Udine, Italy) were used and randomized into six different groups (19 mice/group): one group was treated with PTX vehicle i.v., 1 time/week for 4 weeks and methocel 0.5% os, daily (VEH group); a second group was treated with PTX, i.v., 70 mg/kg 1 time/week for 4 weeks (PTX group) [17]; and the other four groups were treated with PTX, i.v., 70 mg/kg 1 time/week for 4 weeks and co-treated with ricolinostat, p.o., 50 mg/kg daily (PTX + ACY group), or with ITF6475, p.o., 1–6–12.5 mg/kg daily, p.o., (PTX + ITF1, PTX + ITF6, PTX + ITF12.5 groups), respectively (Figure 1). In the days of co-treatment, ricolinostat and all doses of ITF were administered 4 h before PTX. The animals were housed in a certified and limited-access facility with controlled temperature (22 °C ± 2) and humidity (50% ± 20) and an artificial 12 h:12 h light cycle from 7 a.m. to 7 p.m. Mice were monitored daily to evaluate the mortality and their health, and body weight was measured twice a week during the treatment period in order to monitor general conditions and to adjust the dose.

At baseline and at the end of treatment, neurophysiological evaluations and dynamic tests were performed (Figure 1). Baseline neurophysiological results were used to randomize the animals into the 6 groups to ensure homogeneity. At the end of treatment, the animals were sacrificed by isoflurane overdose, caudal nerves, liver, spleen, thymus, kidneys, and sternum were used for histopathological analysis, and skin biopsies for intraepidermal nerve fiber (IENF) density analysis were collected. At the end of the study, blood was collected from the tail vein under isoflurane anesthesia into 1.5 mL Eppendorf tubes, centrifuged at 3500× *g* for 10 min at 4 °C, and then serum was transferred into a new tube and stored at –80 °C until examined for neurofilament light (NfL) level analysis.

### 2.2. Nerve Conduction Studies

The onset of PIPN was assessed by evaluating the sensory nerve conduction velocity (NCV) and nerve action potential amplitude (SNAP) of caudal and digital nerves using the electromyography apparatus (Matrix Light, Micromed, Treviso, Italy). NCV and SNAP were measured by placing a couple of needle recording electrodes (cathode and anode) at the base of the tail (for caudal recordings) or at the ankle bone (for digital recordings) and a couple of stimulating electrodes 3.5 cm away from the recording points (for caudal recordings) or close to the fourth toe (for digital recordings). Latencies were measured from stimulus onset, and peak-to-peak amplitudes were calculated. The NCV was calculated considering the measured distance between the recording and the stimulating negative electrode divided by the latency. The intensity, duration, and frequency of stimulation were set up to obtain optimal results and the maximal amplitude of the potential. All the neurophysiological determinations were performed under standard conditions in a temperature-controlled room (22 ± 2 °C) and the animal under isoflurane anesthesia along the whole procedure with continuous monitoring of vital signs [27,28]. All procedures were performed under deep isoflurane anesthesia while the animal’s body temperature was monitored and kept constant at 37 ± 0.5 °C with a thermal pad electronically connected to a thermal rectal probe (Harvard Apparatus, Holliston, MA, USA).

### 2.3. Pharmacokinetic Assessment

For the determination of ITF6475 in mouse plasma (n. 3 mice/group), aliquots of 20 µL of samples were deproteinized by adding 300 µL of acetonitrile. After centrifugation, 100 µL of the supernatant was collected from each sample and allowed to dry in a nitrogen flow at 35 °C. The residue was then reconstituted in 150 µL of the mixture in 90% water/10% acetonitrile and 0.1% formic acid.

Quantification of ITF6475 in the samples was carried out on a calibration curve in the range 1–1000 ng/mL. Quantification of Ricolinostat in mouse plasma was carried out on aliquots of 20 µL and deproteinized with 200 µL of acetonitrile. Aliquots of 100 µL of the supernatant were collected and diluted with 100 µL of the mixture in 90% water/10% acetonitrile and 0.1% formic acid. The calibration curve range was 1–1000 ng/mL.

Samples were analyzed in an LC-MS/MS system, constituted by a Prominence Shimadzu HPLC (Shimadzu Italia S.r.l., Milano, Italy) and an API6500+ triple quadrupole mass spectrometer equipped with TurboIonSpray (Sciex, Milano, Italy). Analyses were carried out in a positive mode. The system was managed through the software Analyst 1.7.2 (Sciex, Milano, Italy).

### 2.4. Dynamic Aesthesiometer Test

The mechanical nociceptive threshold was assessed using a Dynamic Aesthesiometer Test (model 37450, Ugo Basile Biological Instruments, Comerio, Italy), which generated a linearly increasing mechanical force. At each time point, after the acclimatization period, a servo-controlled mechanical stimulus (a pointed metallic filament, 0.5 mm diameter) was applied to the plantar surface of the hind paw, which exerted a progressively increasing punctuate pressure, reaching up to 15 g within 15 s. The pressure evoking a clear voluntary hind paw withdrawal response was recorded automatically and taken as representing the mechanical nociceptive threshold index. The mechanical threshold was always assessed on alternating sides every 2 min on 3 occasions to yield a mean value. The results represented the maximal pressure (expressed in grams) tolerated by the animals. There was an upper limit cutoff of 20 s, after which the mechanical stimulus was automatically terminated. The examiner (ACa) was blinded regarding the treatment of the animals [27].

### 2.5. Histopathological Analysis

Liver, spleen, thymus, kidneys, and sternum were collected and fixed in 10% buffered formalin at room temperature for 4 days. After fixation, the sternum was decalcified for 48 h at room temperature in 10% buffered EDTA. After trimming, these samples were paraffin-embedded, cut into 3 µm thick sections, stained with hematoxylin–eosin, and examined with an Olympus BX51 light microscope (Olympus, Segrate, Italy).

### 2.6. Neuropathology

Caudal nerves were isolated for morphological analysis and processed as previously described [29], separating the proximal and the distal portions to obtain a better evaluation of the distal-to-proximal extension of the damage. Briefly, the caudal and sciatic nerves were immersion-fixed in 3% glutaraldehyde and were subsequently osmicated, dehydrated, and embedded in epoxy resin. Semithin sections of 1.5 μm thickness were prepared from at least three tissue blocks for each animal, stained with toluidine blue, and examined with a Nexcope Ne920 AUTO light microscope (TiEsseLab Srl, Milano, Italy [27]).

### 2.7. Skin Biopsy

To evaluate the IENF density, glabrous skin punches from the plantar hind paw were fixed in paraformaldehyde-lysine and periodate sodium (PLP) 2%, cryoprotected and serially cut in 20 μm thick sections. Sections were immunostained with rabbit polyclonal anti-protein gene product 9.5 (PGP 9.5; Proteintech, Illinois, Rosemont, IL, USA) using a free-floating protocol. The total number of PGP 9.5-positive IENF crossing the dermal-epidermal junction was counted by a blinded examiner (ACh) under a light microscope at 40× magnification (Nexcope Ne920 AUTO light microscope, TiEsseLab Srl, Milano, Italy). IENF density was expressed as the number of IENFs/length of epidermis (mm) [27].

### 2.8. Serum for NfL Analysis

Serum NfL concentrations were measured using an automated chemiluminescent enzyme immunoassay instrument LUMIPULSE G600 (LUMIPULSE G NfL Blood kit, Fujirebio, Pomezia, Italy). The concentration was expressed in pg/mL [27,30,31].

### 2.9. Statistical Analysis

The numerosity of each group was defined using a power calculation based on the changes in nerve conduction study results, as previously carried out in similar experiments [27]. The differences in body weight, nerve conduction studies, behavioral tests, IENF density, and NfL levels were statistically analyzed with a 2-step approach using a nonparametric one-way Kruskal–Wallis ANOVA test, with Dunn’s post hoc test (with a significance level set at *p* < 0.05). Statistical analyses were performed using the GraphPad Prism4 v.8 statistical package (GraphPad Software, San Diego, CA, USA).

## 3. Results

### 3.1. General Toxicity

**Figure 2:**
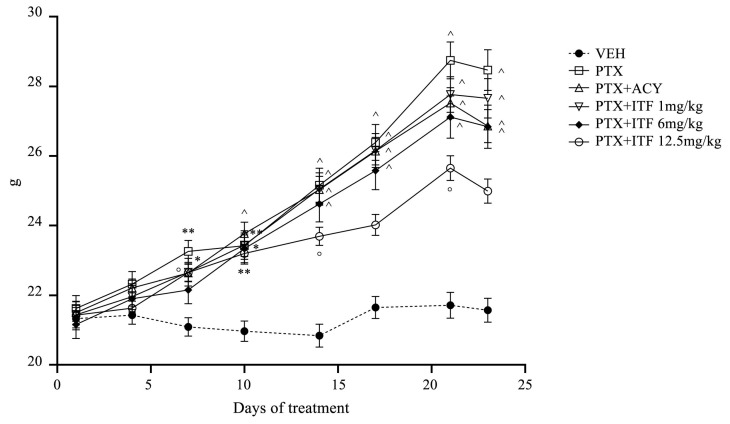
Body weight changes throughout the study. Plots represent the mean value ± SEM. Scheme 0. vs. VEH, * *p* < 0.01 vs. VEH, ** *p* < 0.001 vs. VEH, and ^ *p* < 0.0001 vs. VEH.

The administrations of PTX, ricolinostat, and all doses of ITF6475 were well tolerated by animals, and no mortality was observed. No mice showed evidence of relevant general toxicity. All groups treated with PTX alone or in combination with ricolinosat and all doses of ITF showed a statistically significant increase in body weight vs. VEH (Figure 2).

Histopathological analysis of thymus, spleen, sternal bone marrow, liver, and kidneys showed, at the end of the treatment period, in all the mice examined and treated with PTX alone or in combination with ricolinostat or ITF6475, thymic and splenic hypoplasia associated with reactive extramedullary ectopic hematopoiesis. Mild, diffuse hepatic lipidosis was observed in approximately 25% of the PTX-treated mice examined. At the end of treatment, no pathological findings consequent to treatment were observed in the sternal bone marrow and kidneys.

### 3.2. Pharmacokinetic Assessment

In mouse plasma, ITF6475 is 99% protein-bound (personal observation), while hydroxamic acids such as ricolinostat are not heavily protein-bound.

With this assumption and taking into account that AUCs of ITF6475 increase linearly with dosage, whereas AUCs of ricolinostat increase non-linearly at doses above 25 mg/kg, we can extrapolate that free drug exposures of ITF6475 at the dose of 12.5 mg/kg may be similar to exposures of ricolinostat at the dose of 50 mg/kg.

Table 1 reports the pharmacokinetic results obtained with different doses of ITF6475 and ricolinostat.

### 3.3. Neurotoxicity Assessment

**Figure 3:**
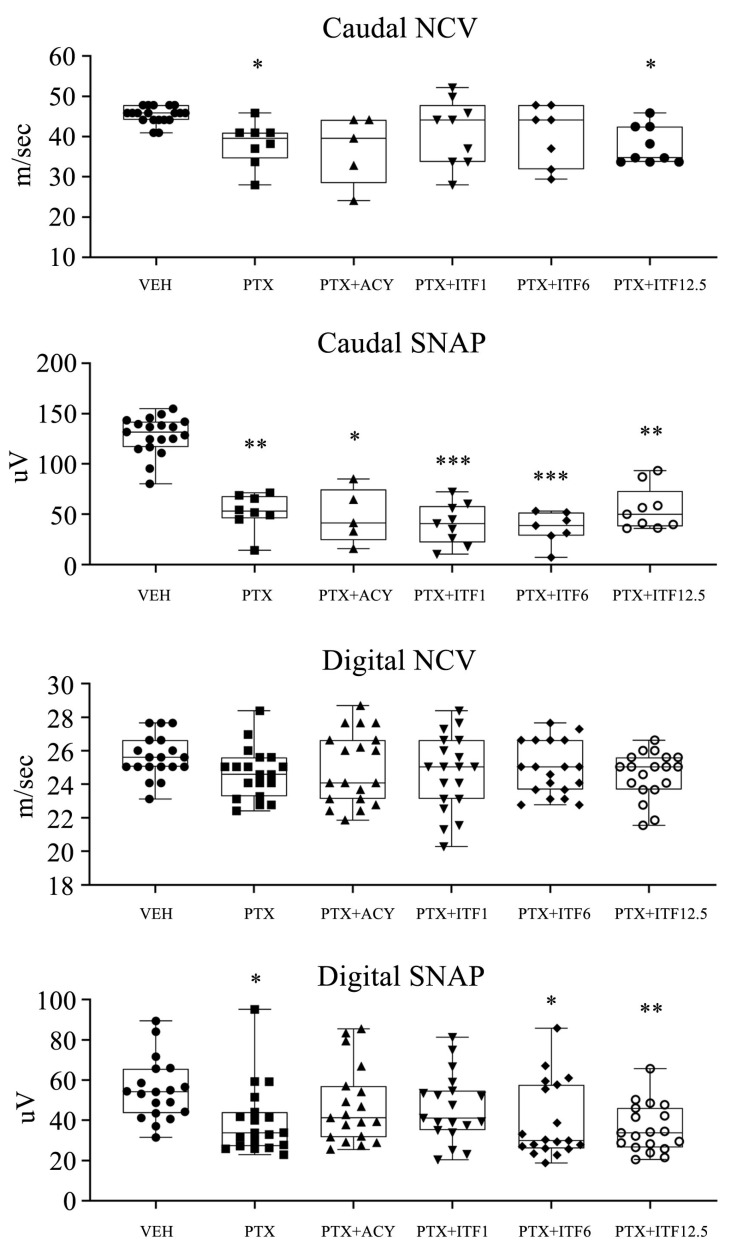
Digital and caudal nerve sensory conduction velocity (NCV) and amplitude (SNAP) at the end of the treatment. Box-and-whisker plots represent the median value, first and third quartiles, and minimum and maximum values. Statistical analysis: nonparametric one-way ANOVA test, Kruskal–Wallis, and Dunn’s post hoc test; * *p* < 0.05 vs. VEH, ** *p* < 0.01 vs. VEH, and *** *p* < 0.0001 vs. VEH.

Neurophysiological evaluation performed at the end of treatment (Figure 3) showed a statistically significant reduction in caudal SNAP in all treated groups compared to VEH.

Only the PTX and PTX + ITF12.5 groups also showed a statistically significant reduction in caudal NCV if compared with VEH. No alterations in digital NCV were observed, while the PTX, PTX + ITF6, and PTX + ITF12.5 groups showed a statistically significant reduction in digital SNAP.

**Figure 4:**
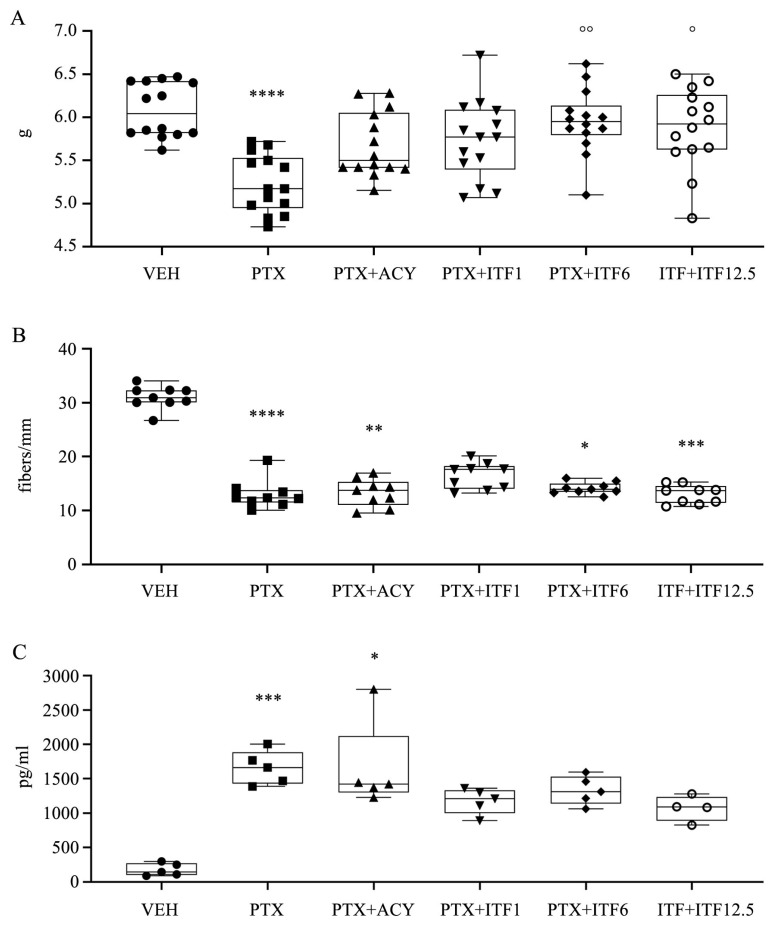
Results at the end of treatment obtained for the Dynamic Aesthesiometer Test (**A**), IENF density (**B**), and NfL levels (**C**). Box-and-whisker plots represent the median value, first and third quartiles, and minimum and maximum values. Statistical analysis: nonparametric one-way ANOVA test, Kruskal–Wallis, and Dunn’s post hoc test; * *p* < 0.05 vs. VEH, ** *p* < 0.01 vs. VEH, *** *p* < 0.001 vs. VEH, **** *p* < 0.0001 vs. VEH, ° *p* < 0.01 vs. PTX, and °° *p* < 0.001 vs. PTX.

At the end of treatment, PTX induced the development of mechanical allodynia as assessed with the Dynamic Aesthesiometer Test (Figure 4A, *p* < 0.0001). At this time point, all the co-treated groups did not show a difference compared to the VEH group, and, in particular, PTX + ITF6 and PTX + ITF12.5 groups showed a statistically significant difference vs. PTX alone (*p* < 0.001 and *p* < 0.01, respectively).

A statistically significant reduction in IENF density was observed in the PTX group compared with the VEH group (*p* < 0.0001) (Figure 4B). Also, PTX + ACY, PTX + ITF6, and PTX + ITF12.5 groups showed a statistically significant reduction (*p* < 0.01, *p* < 0.05, and *p* < 0.001, respectively), while the PTX + ITF1 value was not statistically different from the VEH group.

NfL analysis performed after completion of PTX treatment (Figure 4C), despite a general increase in NfL values, showed a statistically significant difference only in PTX and PTX + ACY groups compared to the VEH group (*p* < 0.001 and *p* < 0.05, respectively), while the increase in the groups co-treated with ITF at any dose was not significant.

**Figure 5:**
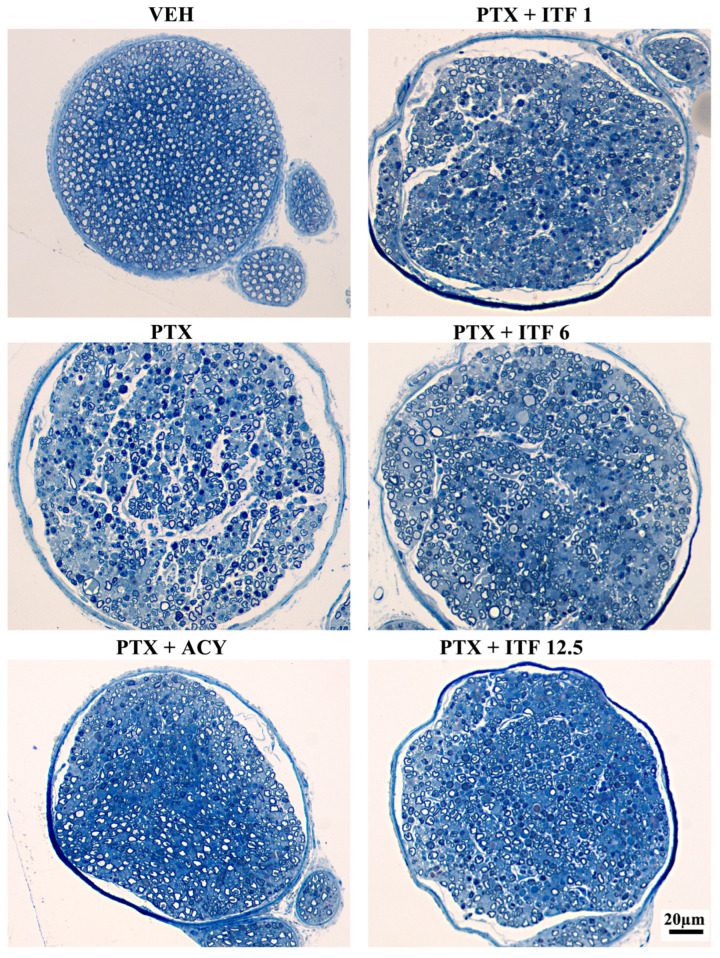
Representative images of proximal caudal nerve samples at the end of treatment.

**Figure 6:**
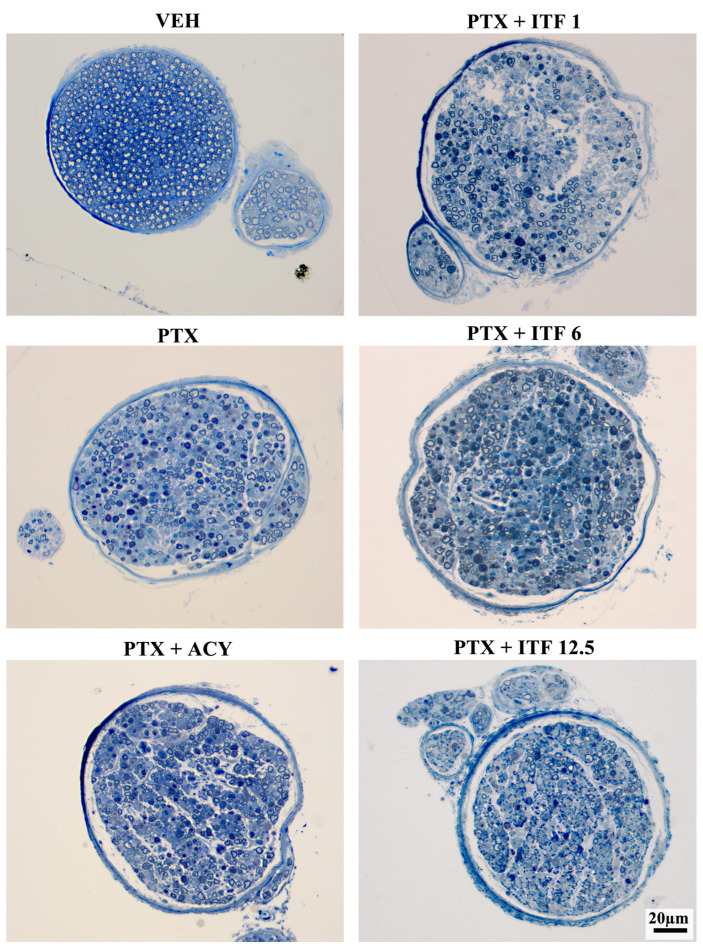
Representative images of distal caudal nerve samples at the end of treatment.

The morphological analysis of the proximal caudal nerves performed at the end of treatment revealed severe axonopathy with numerous degenerated fibers in animals treated with PTX alone or in combination (Figure 5). The morphological analysis of the distal caudal nerves performed at the end of treatment revealed an even more severe axonopathy (Figure 6), which is in agreement with the distal-to-proximal progression of PIPN observed in clinical practice.

## 4. Discussion

The occurrence of CIPN remains a relevant unmet clinical need, with important implications during cancer treatment, and potentially severe, long-term (even permanent) neurological side effects. These side effects may result in impaired sensation and coordination, neuropathic pain, limited dexterity in performing fine manual tasks, impaired balance, and increased risk of falls [7]. The impact of CIPN is so relevant that several attempts have been performed in order to improve its management, but so far, all the pharmacological neuroprotective attempts have failed. These failures suggested the need for a different approach to the prevention of CIPN, supported by evidence-based reasoning. Among these approaches, those based on molecules with absent or minimal risk to interfere with the antineoplastic action of the neurotoxic drugs, and the potential to be highly selective on the peripheral nervous system, are the most promising.

Among putative neuroprotectants with a low risk of reducing anticancer activity of chemotherapeutic drugs, HDAC inhibitors are particularly interesting. In fact, their use has also been suggested as anticancer combination drugs, acting by altering the epigenetic landscape of cancer cells by restoring acetylation and reactivating tumor suppressor genes. These effects might contribute to cycle arrest, to the promotion of cancer cell apoptosis, and, eventually, to the inhibition of cancer cell proliferation. Moreover, the combination of HDAC inhibitors and other chemotherapy drugs could not only exploit the synergistic action of both compounds, but it could also allow the use of lower doses, with a significant decrease in their respective toxicities and a reduction in cancer cell drug resistance.

Acetylation is also a key process in normal cells, being involved in protein folding, autophagy, transcription regulation, signal transduction, differentiation, and neural function, where it acts as a mediator of axonal and neuronal regeneration [32].

Specifically regarding HDAC6, its inhibition has demonstrated efficacy in numerous neuroprotective preclinical studies on both chemotherapy-induced and inherited neuropathies [33]. Notably, some of these studies validated the pharmacological effects of small-molecule inhibitors through gene knockout experiments [34]. This is particularly significant, as many HDAC6 inhibitors lack absolute selectivity [35], making it difficult to rule out the contribution of off-target effects. The alignment between pharmacological and genetic evidence provides strong support for HDAC6’s role in certain neuropathies.

The current understanding of PIPN remains inadequate for effective patient management, with multiple mechanisms proposed [36,37,38]. A key factor appears to be tubulin disruption, leading to cytoskeletal damage and impaired axonal and mitochondrial transport, suggesting a potential neuroprotective role for HDAC6 inhibition [39].

Although the precise mechanism by which HDAC6 inhibition mitigates neuropathy is not fully understood, evidence suggests that this enzyme plays a crucial role in mitochondrial trafficking and energy metabolism. Impaired mitochondrial transport, along with disrupted microtubule dynamics, has been implicated in various neuropathies beyond PIPN [17,40,41,42]. In neurons, mitochondria are transported between the soma and axons via microtubule-associated motor proteins Dynein and Kinesin [43]. HDAC6 destabilizes microtubules by deacetylating α-tubulin [14].

Additionally, HDAC6 interacts with and deacetylates mitochondrial membrane proteins such as Mitochondrial Rho-GTPase (Miro1) and Mitofusin, which are responsible for anchoring mitochondria to motor proteins. This deacetylation weakens mitochondrial attachment, potentially disrupting transport [17]. Under normal conditions, HDAC6 likely functions as a negative regulator of mitochondrial trafficking, and, in cases of neuronal injury, its activity may become even more harmful, impairing neuronal function and regeneration. Beyond its effects on mitochondrial transport, HDAC6 inhibition has been shown to enhance mitochondrial oxygen consumption, glycolysis, and citrate synthesis in DRG neurons [34].

Given prior reports of HDAC6 inhibitors mitigating aspects of CIPN in animal models [16,19,22]—primarily based on behavioral observations—this study employed a comprehensive approach to assess the efficacy of ITF6475 in a well-established mouse model of PIPN. This model implies a very intense PTX treatment through repeated i.v. administration with the aim (i) to mimic the clinical use of the drug as close as possible and (ii) to not only induce a nocifensive behavior in the treated animals, but also severe nerve fiber loss, as it occurs in clinical practice [27]. Despite the high intensity of the PTX treatment, mice did not exhibit any distress signs, their behavior was normal, and the histopathological changes observed in the hematopoietic organs were mild. No pathological changes were observed in the liver and kidney, apart from mild hepatic lipidosis in 25% of the mice. These observations confirm the results of a previous study [27] and rule out the possible bias due to a relevant influence of general toxicity on the neurotoxicity results. Similarly, no evidence of relevant toxicity was observed in the ricolinostat or ITF6475-treated mice.

Our results show that ITF6475 is more effective than ricolinostat, another HDAC6 inhibitor previously evaluated in CIPN models [22]. Daily administration of ITF6475 at 1 mg/kg significantly reduced PTX-induced mechanical allodynia and preserved IENF density. Interestingly, higher doses led to a reduced protective effect, for reasons that remain unclear and warrant further investigation.

The neuroprotective effect of ITF6475 was further supported by changes in NfL levels at the end of treatment. While all PTX-treated groups exhibited significant NfL elevations, those receiving ITF6475 had considerably lower increases compared to mice treated with PTX alone or in combination with ricolinostat. This observation, together with the significant protection of reduction in digital nerve SNAP induced by PTX administration achieved by the co-administration of ricolinostat and ITF6475 at the 1 mg/kg dose, suggests that a positive effect was observed on large myelinated fibers, although it seems that small fibers are more effectively protected, based on behavioral and IENF density assessments. This finding is largely consistent with previous studies on HDAC6 inhibitors, which focused on less severe neurotoxic exposure and lacked detailed pathological assessments of myelinated fibers. Nonetheless, these studies consistently demonstrated that HDAC6 inhibitors alleviate small fiber neuropathy and neuropathic pain, as evidenced by behavioral assessments and IENF density measurements.

In conclusion, our findings support the hypothesis that HDAC6 inhibitors, particularly ITF6475, can effectively prevent small fiber damage in a severe, chronic PIPN model, as demonstrated through behavioral, pathological, and serological evaluations. The difference in the neuroprotective efficacy between small and large myelinated fibers may stem from the severity of nerve damage in our PIPN model, which involves prolonged high-dose PTX exposure. Alternatively, it could indicate a selective efficacy of this neuroprotective strategy.

## Figures and Tables

**Table 1:** Pharmacokinetic results.

Ricolinostat		
Dose	AUC_0–8h0–8h00_ (ng·h/mL)	Cmax (ng/mL)
25 mpk	124	33.7
100 mpk	171	29.8
200 mpk	234	33.9
**ITF6475**		
**Dose**	**AUC_0–24h0–8h00_ (ng·h/mL)**	**Cmax (ng/mL)**
25 mpk	28,958	4328
100 mpk	157,797	14,631
200 mpk	271,434	22,485

## Data Availability

The datasets used and analyzed during the current study contain confidential information, but they are available from the corresponding author upon reasonable request and under a non-disclosure agreement.

## Abbreviations

The following abbreviations are used in this manuscript:

CIPN	chemotherapy-induced neuropathy
DMSO	dimethyl sulfoxide
EDTA	ethylenediaminetetraacetic acid
GTPase	guanosine triphosphate hydrolase
HDAC	histone deacetylase
IENF	intraepidermal nerve fibers
i.v.	intravenous
mpk	mg/kg
NCV	sensory nerve conduction velocity
PEG	polyethylene glycol
PGP 9.5	protein gene product 9.5
PIPN	paclitaxel-induced peripheral neuropathy
PLP	paraformaldehyde-lysine and periodate sodium
p.o.	oral
PTX	paclitaxel
SNAP	sensory nerve action potential amplitude
VEH	paclitaxel vehicle
